# Der Einsatz von Immuncheckpoint-Inhibitoren im onkologischen Alltag

**DOI:** 10.1007/s00393-020-00876-2

**Published:** 2020-09-16

**Authors:** Julian Schardt

**Affiliations:** Universitätsklinik für Medizinische Onkologie, Inselspital, Freiburgstr. 41G, 3010 Bern, Schweiz

**Keywords:** Immunvervittelte Nebenwirkungen, Anti-PD1, Anti-CTLA4, Ipilimumab, Nivolumab, Immune-mediated adverse events, Anti-PD1, Anti-CTLA4, Ipilimumab, Nivolumab

## Abstract

**Hintergrund:**

Die Einführung von Immuncheckpoint-Inhibitoren (ICI) hat die Behandlungskonzepte der Onkologie für eine Vielzahl von unterschiedlichen Krebsarten maßgeblich verändert. Dabei werden in der klinischen Routine v. a. humanisierte Antikörper gegen Immuncheckpoints wie „cytotoxic T‑lymphocyte associated protein 4“ (CTLA-4) oder „programmed cell death 1/programmed cell death ligand 1“ (PD1/PD-L1) eingesetzt.

**Fragestellung:**

Übersicht zur Therapielandschaft mit Immuncheckpoint-Inhibitoren bei mehrheitlich soliden Tumoren in der Onkologie.

**Material und Methoden:**

Darstellung und Diskussion aktueller Studienresultate, Einbezug aktueller Behandlungsempfehlungen und Zulassungsindikationen.

**Ergebnisse:**

Sieben verschiedene Immuncheckpoint-Inhibitoren werden in der Onkologie therapeutisch eingesetzt: ein Anti-CTLA-4-Antikörper, 3 Anti-PD1-Antikörper und 3 Anti-PD-L1-Antiköper. FDA-Zulassung auf dem US-Markt für 17 verschiedene Tumorentitäten und einer agnostischen Indikation (Tumoren mit defizienter Mismatch-repair-Maschinerie/hohe Mikrosatelliteninstabilität). Langzeitremissionen sind in ca. zwei Drittel der Patienten mit Tumoransprechen möglich.

**Schlussfolgerungen:**

Nutzen der Immuncheckpoint-Inhibitoren nur für einen Teil der behandelten Patienten. Primäre und sekundäre Resistenzmechanismen erst in Anfängen verstanden. Kombinationstherapien der Immuncheckpoint-Inhibitoren mit z. B. Chemotherapie, neuen Immuncheckpoint-Inhibitoren (z. B. Anti-LAG3-Antikörper) oder gezielten Therapien (z. B. CDK4/6, PARP-Inhibitoren) zur Verbesserung der Wirksamkeit werden in klinischen Studien untersucht. Verlässliche, prädiktive Marker sind dringend erforderlich.

## Abstract (en)

**Background:**

The advent of immune checkpoint inhibitors has dramatically changed the treatment landscape of many different tumor types. In the clinical routine, humanized antibodies against the immune checkpoints cytotoxic T‑lymphocyte associated protein 4 (CTLA-4) and programmed cell death 1/programmed cell death ligand 1 (PD1/PD-L1) are generally applied.

**Objective:**

This article provides an overview of the treatment landscape with immune checkpoint inhibitors, especially for solid tumors in oncology.

**Material and methods:**

Description and discussion of recent trial results as well as current treatment recommendations and treatment approval indications.

**Results:**

In the clinical routine seven different immune checkpoint inhibitors are applied in oncology: one anti-CTLA‑4 antibody, three anti-PD1 antibodies and three anti-PD-L1 antibodies. On the US market FDA approval for 17 different tumor entities and one agnostic indication (tumors with mismatch repair mechanism deficiency/high microsatellite instability). Long-term remission can be achieved for ca. two thirds of patients with tumor response.

**Conclusion:**

Clinical benefits for only a part of patients treated with immune checkpoint inhibitors. The understanding of primary and secondary mechanisms of resistance to immune checkpoint inhibitors has just begun to evolve. Combination strategies of immune checkpoint inhibitors with e.g. chemotherapy, new immune checkpoint inhibitors (e.g. anti-LAG3 antibody) or targeted therapies (e.g. CDK4/6, PARP inhibitors) to improve efficacy are under clinical investigation. Reliable and predictive biomarkers are urgently needed.

Die Entwicklung und Einführung von Immuncheckpoint-Inhibitoren (ICI) hat in den vergangenen Jahren die Behandlungskonzepte der Onkologie für eine Vielzahl von unterschiedlichen Krebsarten aus dem Bereich der soliden Tumoren wie auch einiger hämatologischer Krebserkrankungen maßgeblich verändert. Dabei werden in der klinischen Routine bis dato v. a. humanisierte Antikörper gegen Immuncheckpoints wie „cytotoxic T‑lymphocyte associated protein 4“ (CTLA-4) oder „programmed cell death 1/programmed cell death ligand 1“ (PD1/PD-L1) eingesetzt. Im Gegensatz zu den konventionellen Chemotherapeutika, oder gezielten („targeted“) Therapien aus dem Feld der personalisierten Medizin (z. B. Tyrosinkinase-Inhibitoren), die mehrheitlich einen direkten Effekt auf die Krebszellen ausüben, wirken die ICI indirekt durch Aktivierung des körpereigenen Immunsystems, v. a. tumorspezifischer, zytotoxischer T‑Lymphozyten. Wissenschaftliche Innovation gepaart mit einer wachsenden wirtschaftlichen Bedeutung für die pharmazeutische Industrie spiegeln sich in der stetig wachsenden Zahl von klinischen Studien mit unterschiedlichen Antikörpern insbesondere zur Blockierung der PD1/PD-L1-Achse wider: So werden aktuell (Stand Oktober 2019) global mehr als 70 verschiedene Zielmoleküle, die auf die T‑Zell-Aktivität modulierend wirken, in über 3400 klinischen Studien auf deren Verträglichkeit und Wirksamkeit evaluiert [1]. Im folgenden Artikel sollen die relevanten Tumorentitäten, in denen ICI bereits mit Erfolg im klinischen Alltag eingesetzt werden, näher vorgestellt werden.

## Immuncheckpoint-Inhibitoren im klinischen Alltag

Die erste Zulassung eines ICI erfolgte im März 2011 für den Anti-CTLA-4-Antikörper Ipilimumab für Patienten mit nichtresezierbarem und/oder metastasiertem malignem Melanom und markiert gleichzeitig den Beginn einer sich rasch entwickelnden neuen Generation an Onkologika, den Immuntherapien. Ipilimumab war die erste Substanz, die einen signifikanten Überlebensvorteil der Patienten mit metastasiertem Melanom gegenüber der damaligen Standardtherapie mit Dacarbazin zeigte, mit 22 % Langzeitüberlebenden und einer für ICI häufig zu beobachtenden, charakteristischen Plateaubildung der Überlebenskurve im Falle des metastasierten Melanoms nach 3 Jahren.

Seit der Zulassung für Ipilimumab hat sich die Behandlungslandschaft in der Onkologie rasant verändert [2]. Insbesondere Antikörper zur Blockierung der PD1/PD-L1-Achse, die ein besseres Wirkungs- und günstigeres Nebenwirkungsprofil als die Anti-CTLA-4-Antikörper in der Monotherapie aufweisen, finden heute Anwendung im klinischen Alltag.

Die meisten Zulassungsindikationen erfolgten bisher fast ausschließlich für fortgeschrittene (nichtresezierbare oder metastasierte), solide Tumoren. Dabei wurden die ICI zunächst in späteren Therapielinien (nach Versagen der Standardtherapie wie z. B. Dacarbazin für das metastasierte Melanom oder einer platinhaltigen Kombinationstherapie für das nichtkleinzellige Bronchialkarzinom) untersucht.

Erst jüngere Entwicklungen untersuchen die Wirksamkeit von ICI als Monotherapie, oder als Kombinationspartner mit z. B. einer konventionellen Chemotherapie in der Erstlinienbehandlung im metastasierten Stadium wie auch im neoadjuvanten- und adjuvanten Setting (z. B. beim Melanom, Brustkrebs oder Blasenkrebs).

Bis heute (Stand Oktober 2019) wurden insgesamt 7 ICI von der FDA zugelassen (Tab. 1). Dabei handelt es sich um humanisierte, monoklonale Antikörper die zum einen gegen CTLA‑4 (Ipilimumab) gerichtet sind, sowie je 3 Antikörper die PD1 (Pembrolizumab, Nivolumab, Cemiplimab) oder PD-L1 (Atezolizumab, Durvalumab, Avelumab) als Zielmolekül aufweisen.

**Table 1:** 

Wirkstoff	Handelsname	Target	Klasse
Ipilimumab	Yervoy® (Bristol Myers Squibb, USA)	CTLA4	Humaner IgG1-Antikörper
Nivolumab	Opdivo® (Bristol Myers Squibb, USA)	PD1	Humaner IgG4-Antikörper
Pembrolizumab	Keytruda® (Merck, USA)	PD1	Humaner IgG4-Antikörper
Cemiplimab	Liptayo (Sanofi, Frankreich)	PD1	Humaner IgG4-Antikörper
Atezolizumab	Tecentriq® (Roche, Schweiz)	PD-L1	Humaner IgG1-Antikörper
Durvalumab	Imfinzi® (Astra Zeneca, UK)	PD-L1	Humaner IgG1-Antikörper
Avelumab	Bavencio® (Merck Serono, USA)	PD-L1	Humaner IgG1-Antikörper

*CTLA4* „cytotoxic T‑lymphocyte associated protein 4“, *PD1* „programmed cell death protein 1“, *PD-L1* „programmed cell death protein ligand 1“

**Figure 1:**
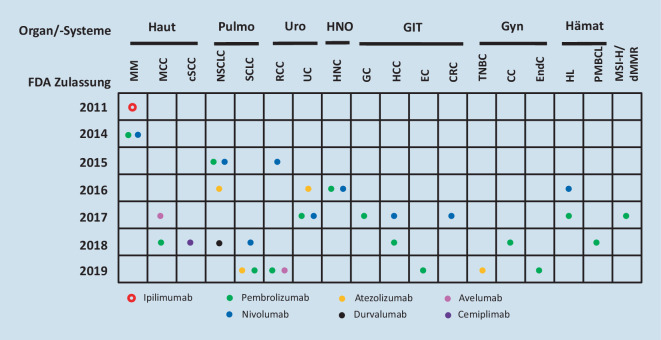

Die hohe Studienaktivität im Bereich der Immuntherapeutika findet einerseits ihren Ausdruck in der raschen Zunahme und Ausweitung der Indikationen einzelner ICI (insgesamt 17 Zulassungen seit 2011, davon allein 13 in den vergangenen 2 Jahren), führt aber auch andererseits dazu, dass gleich mehrere ICI in derselben Indikation (z. B. Atezolizumab, Nivolumab und Pembrolizumab als Zweitlinienbehandlung für das metastasierte Urothelkarzinom) die Zulassung erhielten. In Abb. 1 sind die unterschiedlichen Tumorentitäten, die entsprechend zur Behandlung zugelassenen ICI sowie deren Zulassungsjahr auf dem U.S.-Markt durch die FDA veranschaulicht dargestellt.

Zwei wesentliche Charakteristika der neuen Therapien mit ICI bestehen (i) in der Ausbildung eines immunologischen Gedächtnisses mit für einen Teil der Patienten lang anhaltenden Remissionen und (ii) dem Auftreten eines neuen Spektrums an Nebenwirkungen, sog. immunvermittelten Nebenwirkungen, welche sich als autoimmune Phänomene manifestieren und jedes Organ betreffen können, wie z. B. Arthritiden, Kolitiden, Hepatitis oder Endokrinopathien.

Die im klinischen Alltag zu beobachtenden, erstaunlichen Langzeitremissionen für auch aggressive Tumoren wie dem metastasierten malignen Melanom (5-Jahres-Gesamtüberleben von 52 %) treffen bis heute aber nur für einen Teil der behandelten Patienten zu, sodass auch für die ICI primäre und sekundäre Resistenzmechanismen über den Therapieerfolg entscheiden. Die Mechanismen zur Ausbildung von Resistenzen unter einer ICI-Therapie werden aber erst in Ansätzen verstanden (z. B. Immunoediting der Krebszellen mit Verlust von Neoantigenen, Koexpression von T‑Zell-inhibierenden Rezeptoren [u. a. LAG‑3, TIM-3] oder aber die Präsenz von „myeloid-derived suppressor cells“ im Tumorgewebe [3]).

Im Folgenden Teil des Artikels sollen die Indikationen zum Einsatz der ICI im klinischen Alltag, sowie die relevanten klinischen Daten, die zur Zulassung führten, für die einzelnen Tumorentitäten genauer besprochen werden. An dieser Stelle sei darauf hingewiesen, dass die Indikationen für den Einsatz der ICI beim malignen Melanom in einem separaten Beitrag dieser Ausgabe behandelt werden. Der folgende Artikel klammert daher diese spezifische Tumorentität mehrheitlich von der Besprechung aus.

### Plattenepithelkarzinome der Haut und Merkel-Zell-Karzinome

Metastasierte Plattenepithelkarzinome der Haut bei Patienten ohne langjährige Immunsuppression sind im klinischen Alltag eine vergleichsweise seltene Entität. Für diese Patientengruppe steht mit dem Anti-PD1-Antikörper Cemiplimab eine neue, vielversprechende Therapiemöglichkeit mit hohen Ansprechraten (ca. 50 %, davon 17 % komplette Remissionen) zur Verfügung [4]. Über 60 % der Patienten mit einem radiologisch dokumentierten Ansprechen erzielten eine längerfristige Remission mit einem medianen progressionsfreien Überleben von mindestens 18 Monaten. Das mediane Gesamtüberleben dieser Studienpatienten ist bei Fertigstellung dieses Manuskripts noch nicht erreicht gewesen.

Gute Erfolge werden auch für behandlungsnaive Patienten mit lokal fortgeschrittenem oder metastasiertem Merkel-Zell-Karzinom unter einer Behandlung mit dem Anti-PD1-Antikörper Pembrolizumab oder auch dem Anti-PD-L1-Antikörper Avelumab beobachtet mit rund 60 % Ansprechrate. Mehr als zwei Drittel der Patienten mit radiologisch dokumentiertem Tumoransprechen erzielten längerfristige Remissionen (komplette oder partielle) von über 6 Monaten [5, 6].

### Nichtkleinzelliges und kleinzelliges Bronchialkarzinom

Neben dem malignen Melanom steht das primäre Bronchialkarzinom ähnlich stark im Fokus klinischer Studien mit ICI v. a. gegen die PD1/PD-L1-Achse.

Zunächst wurde dieser Therapieansatz beim metastasierten NSCLC in der zweiten Therapielinie nach Versagen einer vorhergehenden zytotoxischen Chemotherapie zugelassen. Dabei zeigte sich in 2 Phase-I-/II-Studien für den Einsatz von Nivolumab gegenüber der Zweitlinienchemotherapie mit Docetaxel ein signifikanter 5‑Jahres-Überlebensvorteil von 13,4 % vs. 2,6 % [7]. Phase-I-/II-Studien

**Figure 2:**
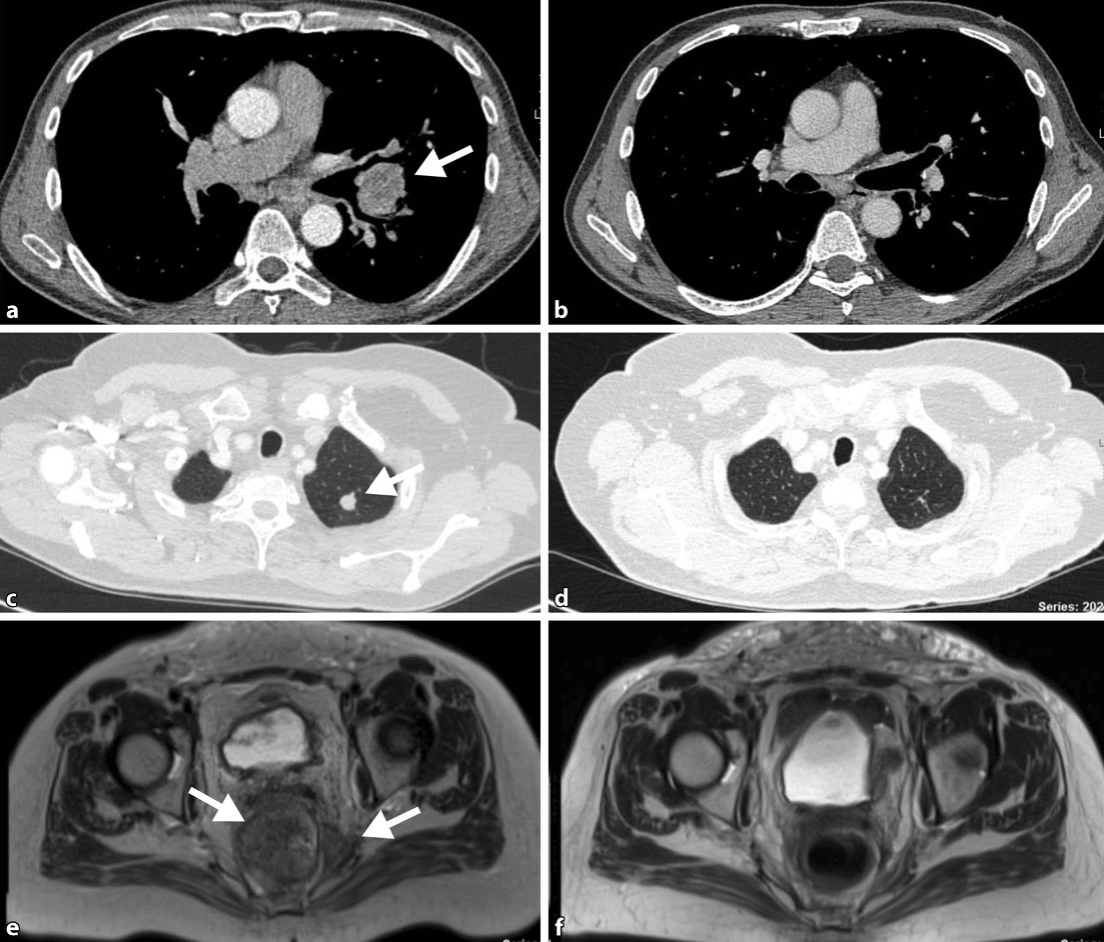

Aktuelle klinische Studien belegen indes auch Erfolge der Anti-PD1-Blockierung in der ersten Therapielinie: So ist eine Kombination des Anti-PD1-Antikörpers Pembrolizumab mit Carboplatin und Pemetrexed (NSCLC: Typ Adenokarzinom) oder Carboplatin und Paclitaxel (NSCLC: Typ Plattenepithelkarzinom) einer alleinigen Chemotherapie hinsichtlich des Gesamtüberlebens der Patienten signifikant überlegen: 69,2 % vs. 49,4 % nach 12 Monaten für Adenokarzinome und median 15,9 Monate vs. 11,3 Monate für Plattenepithelkarzinome der Lunge [8, 9]. Als ausgesprochen wirksam stellte sich die alleinige Behandlung mit Pembrolizumab für Lungenkarzinome mit einer PD-L1-Expression von ≥50 % der Tumorzellen heraus (ca. 25 % der fortgeschrittenen Bronchialkarzinome weisen diese hohe Expression auf): Die Ansprechraten lagen in der Studie bei rund 45 % vs. 28 % unter alleiniger Chemotherapie und führten zu einem deutlich verbesserten Überleben (medianes Gesamtüberleben 30,5 Monate vs. 14,2 Monate) bei gleichzeitig besserer Verträglichkeit der Immuntherapie gegenüber der konventionellen Chemotherapie [10]. Die Abb. 2a, b zeigt exemplarisch den Verlauf des Primarius (NSCLC) eines 62-jährigen Patienten mit metastasiertem NSCLC (PD-L1-Expression 80 % der Tumorzellen) unter Behandlung mit Pembrolizumab über einen Zeitraum von 30 Monaten (stabile partielle Remission).

Für Patienten mit einem nichtresezierbaren, lokal fortgeschrittenen Bronchialkarzinom (Stadium III), deren Erkrankung unter einer primären, kombinierten Radiochemotherapie keine Progression zeigte, ist die konsolidierende Weiterbehandlung mit dem Anti-PD-L1-Antiköper Durvalumab für insgesamt 12 Monate mit einer eindrücklichen Verbesserung des medianen progressionsfreien Überlebens verbunden (16,8 Monate vs. 5,6 Monate) [11].

Auch für das kleinzellige Bronchialkarzinom im palliativen Setting („extensive disease“) hat in der ersten Therapielinie die Zugabe des Anti-PD-L1-Antikörpers Atezolizumab zu einer Standardchemotherapie mit Carboplatin und Etoposid einen moderaten, wenn auch signifikanten Überlebensvorteil gegenüber der bisherigen Therapie mit Carboplatin/Etoposid gezeigt (medianes Gesamtüberleben 12,3 vs. 10,3 Monaten) und sich im klinischen Alltag als neuer Standard etabliert [12].

### Urogenitale Tumore

Innerhalb dieser großen Gruppe von sehr unterschiedlichen Tumoren (Hodentumoren, Niere‑, Blase‑, Prostatakarzinom) sind es bisher v. a. Patienten mit klarzelligem Nierenzellkarzinom und Patienten mit urothelialen Karzinomen, die von einer Immuntherapie mit ICI profitieren können:

Metastasierte Nierenzellkarzinome weisen einen sehr heterogenen Krankheitsverlauf auf mit einem medianen Überleben zwischen 7,5 Monaten („poor risk“) und 43 Monaten („favorable risk)“ je nach Risikostratifizierung (Heng-Score) [13]. Die meisten Patienten wurden bisher unabhängig von ihrer Risikogruppierung in der ersten, palliativen Therapielinie mit einem Tyrosinkinase-Hemmer (Sunitinib oder Pazopanib) behandelt. Bei intermediärem sowie ungünstigem Risikoprofil („intermediate/poor risk group“) konnte aber mit der ICI-Kombination Ipilimumab/Nivolumab neben deutlich höheren Ansprechraten (41 % vs. 34 %) auch ein signifikant verlängertes Gesamtüberleben (64 % vs. 56 % nach 30 Beobachtungsmonaten) gegenüber der alleinigen Behandlung mit dem Tyrosinkinase-Hemmer Sunitinib erzielt werden [14]. Für Patienten mit metastasiertem, klarzelligem Nierenzellkarzinom und einem ungünstigem („intermediate/poor risk“) Risikoprofil gilt die ICI-Kombination heute als die Therapie der Wahl in der ersten Linie.

Neue Therapieansätze in der Erstlinienbehandlung werden mit einer Kombination eines ICI (Avelumab oder Pembrolizumab) und dem Tyrosinkinase-Hemmer Axitinib verfolgt: Unabhängig von einer Einteilung der Patienten zu einer der prognostischen Risikogruppen zeigten diese Kombinationstherapien sehr hohe Ansprechraten (55–59 %) sowie ein signifikant verlängertes progressionsfreies Überleben im Vergleich zu einer alleinigen Behandlung mit dem Tyrosinkinase-Hemmer Sunitinib (15,1 Monate vs. 11,1 Monate für Pembrolizumab + Axitinib vs. Sunitinib; 13,8 Monate vs. 8,4 Monate für Avelumab + Axitinib vs. Avelumab) [15, 16]. Beide Kombinationstherapien stellen einen neuen Standard in der Erstlinienbehandlung des metastasierten Nierenzellkarzinoms für Patienten mit günstiger Prognose („favorable risk“) dar.

Für fortgeschrittene/metastasierte Urothelkarzinome der Blase hat sich nach Versagen einer Erstlinientherapie mit einer platinhaltigen Chemotherapie (Cisplatin + Gemcitabin oder Carboplatin + Gemcitabin) die Behandlung mit einem ICI, der die PD1/PD-L1-Achse blockiert, etabliert: In den entsprechenden Studien zeigten sich zwar nur moderate Ansprechraten (rund 20 %), doch für etwa zwei Drittel der Patienten mit einem Tumoransprechen können zum Teil langfristige Remissionen bei guter Lebensqualität erzielt werden [17–19]. Die Abb. 2c, d zeigt beispielhaft den Verlauf einer Lungenmetastase, ausgehend von einem Urothelkarzinom der Blase einer 72-jährigen Patientin unter einer Therapie mit Pembrolizumab mit stabiler Remission seit 21 Monaten.

Wie für das Bronchialkarzinom oder das Nierenzellkarzinom wird auch beim Blasenkarzinom die ICI-Therapie nach Erfolgen in der zweiten Therapielinie als Erstlinienbehandlung untersucht: Für die Kombinationsbehandlung von Atezolizumab (Anti-PD-L1-Antikörper) mit einer Standardchemotherapie (Cisplatin/Gemcitabin oder Carboplatin/Gemcitabin) konnte das mediane progressionsfreie Überleben gegenüber der alleinigen platinbasierten Chemotherapie signifikant, wenn auch nur moderat von 6,3 Monaten auf 8,2 Monate verbessert werden. Die Ergebnisse für das Gesamtüberleben aus der Studie sind aktuell aber noch nicht reif genug, um eine definitive Aussage über den Stellenwert der Kombinationstherapie (ICI + Chemotherapie) treffen zu können [20].

### Fortgeschrittene Plattenepithelkarzinome im HNO-Bereich

Der allgemeine Trend der pharmazeutischen Industrie, die palliative Therapie mit ICI in die Erstlinienbehandlung zu integrieren, lässt sich auch anhand der fortgeschrittenen HNO-Tumoren verfolgen: Für rezidivierte oder metastasierte Plattenepithelkarzinome des Kopf-Hals-Bereiches hatte sich zunächst die Behandlung mit Nivolumab als Zweitlinientherapie etabliert bei nachweislich signifikant verbessertem Gesamtüberleben unter einer Monotherapie mit Nivolumab gegenüber einer konventionellen Chemotherapie (7,5 Monate vs. 5,1 Monate im Median) [21].

In der Folge untersuchte eine dreiarmige Phase-III-Studie die Wirksamkeit des Anti-PD1-Antikörpers Pembrolizumab als alleinige Therapie oder als Kombinationspartner zu einer konventionellen Chemotherapie (Cisplatin/5-Fluorouracil oder Carboplatin/5-Fluorouracil) im Vergleich zu dem bisherigen Standard, einer Kombination aus Erbitux + konventioneller Chemotherapie (Cisplatin/5-Fluorouracil oder Carboplatin/5-Fluorouracil) [22]: Für Patienten, deren Tumor immunhistochemisch eine erhöhte PD-L1-Expression aufwies („combined positive score“ [CPS] ≥1), zeigte die alleinige Behandlung mit Pembrolizumab einen signifikanten Überlebensvorteil im Vergleich zur Standardtherapie. Pembrolizumab, kombiniert mit einer konventionellen Chemotherapie, führte ebenfalls zu einem verbesserten Gesamtüberleben unabhängig von dem PD-L1-CPS-Score. Im klinischen Alltag wird daher die Monotherapie mit Pembrolizumab für Patienten mit rezidiviertem oder metastasiertem Plattenepithelkarzinom im HNO-Bereich und erhöhter PD-L1-Expression (CPS ≥1) als neuer Standard in der Erstlinienbehandlung angesehen. Für die Subgruppe der Patienten mit einem CPS von <1 wird die Kombination aus Pembrolizumab + konventioneller Chemotherapie eingesetzt.

### Gastrointestinale Tumoren und das hepatozelluläre Karzinom

In der Behandlung von Malignomen des Gastrointestinaltraktes erwiesen sich die ICI-Therapien bisher als nur wenig wirksam, Ausnahmen finden sich aber für molekular vordefinierte Patientengruppen:

So zeigte zwar eine Behandlung des metastasierten Adenokarzinoms des Magens und des 2 Vortherapien hatten, bescheidene Ansprechraten von 11 %, sowie eine Verlängerung des medianen Überlebens von 4,1 Monaten (mit Placebo behandelte Patienten) auf lediglich 5,3 Monate [23].

Ein kleiner, jedoch signifikanter Überlebensvorteil wurde aber kürzlich durch die Behandlung mit Pembrolizumab von Patienten mit vorbehandelten Ösophaguskarzinomen und einer hohen PD-L1-Expression des Tumors gezeigt („combined positive score“ [CPS] ≥10) [24]: Für diese molekulare Subgruppe an Patienten betrug das Gesamtüberleben für die mit Pembrolizumab behandelten Patienten 9,3 Monate vs. 6,7 Monate unter konventioneller Chemotherapie. Basierend auf diesen Phase-III-Studiendaten sowie den Ergebnissen einer Vorläuferstudie, erteilte die FDA im Juli 2019 die Zulassung von Pembrolizumab für diese molekular vordefinierte Patientenpopulation [24, 25].

Vergleichsweise hohe Ansprechraten werden auch für die kleine Gruppe der metastasierten, kolorektalen Karzinome beobachtet, deren Tumoren eine hohe Mikrosatelliteninstabilität als Ausdruck einer defizienten Mismatch-repair-Maschinerie (dMMR) aufweisen: Unter einer Behandlung mit Nivolumab als Monotherapie oder in Kombination mit Ipilimumab konnten Ansprechraten von 31–54 % mit zum Teil lang anhaltenden Remissionen erzielt werden [26, 27].

In der Behandlung des fortgeschrittenen oder metastasierten, hepatozellulären Karzinoms spielen Therapien gegen die PD1/PD-L1-Achse heute eine zentrale Rolle für Patienten, die bereits mit dem Tyrosinkinase-Hemmer Sorafenib vorbehandelt wurden: Nivolumab (CheckMate 040-Studie) und Pembrolizumab (Keynote-224-Studie) erzielten in Studien Ansprechraten von rund 20 % und ca. zwei Drittel der Patienten mit einem dokumentierten Tumoransprechen konnten stabile Remissionen über mindestens 9 Monate hinaus erreichen [28, 29].

### Gynäkologische Tumoren

ICI haben erst kürzlich Zulassungen für Indikationen aus dem gynäkoonkologischen Bereich erhalten und dies primär für molekular vordefinierte Patientensubgruppen.

Die Behandlung mit ICI für Brustkrebspatientinnen zeigte in erster Linie für das metastasierte, triple-negative Mammakarzinom (immunhistochemisch fehlende Expression von Östrogen‑, Progesteronrezeptor und HER2) in Kombination mit einer Chemotherapie einen klinischen Nutzen: Während mit einer Behandlung als Monotherapie (Atezolizumab) lediglich ein Ansprechen (partielle Remission + komplette Remissionen) von 10 % erreicht wurde, konnte in der Kombination von Atezolizumab mit Nab-Paclitaxel als Erstlinienbehandlung im Vergleich zu Nab-Paclitaxel als Monotherapie ein Ansprechen von 56 % (vs. 45,9 % Nab-Paclitaxel-Monotherapie) sowie ein signifikant verbessertes progressionsfreie Überleben (7,2 vs. 5,5 Monate Nab-Paclitaxel-Monotherapie) und verbessertes Gesamtüberleben (21,3 vs. 17,6 Monate) erzielt werden. Für die Subgruppe der Patientinnen mit PD-L1-positiven Tumoren (PD-L1-Expression auf tumorinfiltrierenden Immunzellen ≥1 %) ließ sich für die Kombinationsbehandlung der größte Nutzen hinsichtlich des medianen Gesamtüberlebens gegenüber der alleinigen Chemotherapie erzielen: 25 vs. 15,5 Monate [30, 31].

In einer Phase-II-Studie wurden Patienten mit unterschiedlichen soliden Tumoren im fortgeschrittenen Stadium, die zumindest eine etablierte Therapie vorgängig erhalten hatten, mit Pembrolizumab in fixer Dosierung von 200 mg alle 3 Wochen für maximal 2 Jahre behandelt. In diesem Mix an Tumoren finden sich so unterschiedliche Tumorentitäten wie Endometriumkarzinome, Vulvakarzinome, Zervixkarzinome und solide Tumoren mit nachweislich hoher Mikrosatelliteninstabilität (MSI-H). Aus dieser Studie wurden kürzlich die Interimsresultate für Patienten mit vorbehandeltem und fortgeschrittenem Zervixkarzinom publiziert: 12 von 98 behandelten Patienten (12,2 %) zeigten ein Tumoransprechen (3 komplette und 9 partielle Remissionen), die mediane Dauer des Ansprechens ist zum aktuellen Zeitpunkt noch nicht erreicht gewesen (≥3,7 Monate bis ≥18,6 Monate) [32]. Für die Tumoren der Patienten mit einem Ansprechen ließ sich immunhistochemisch eine Positivität für PD-L1 („combined positive score“ [CPS] ≥1) nachweisen, sodass in dem Zulassungstext der FDA der Einsatz von Pembrolizumab für das Zervixkarzinom auf Tumoren mit einem CPS ≥1 beschränkt wurde.

Die therapeutischen Möglichkeiten für Patientinnen mit einem rezidivierten Endometriumkarzinom, die bereits eine systemische Behandlung mit Carboplatin/Taxol (adjuvant oder als Erstlinienbehandlung) erhalten hatten, sind nur noch sehr begrenzt. Ein vielversprechender therapeutischer Ansatz für diese Patientinnen wurde mit der Kombination des ICI Pembrolizumab und dem oralen Multi-Tyrosinkinase-Hemmer Lenvatinib (Zielmoleküle: u. a. VEGFR 1–3, FGFR 1–4, PDGFR-alpha, RET, KIT) im Rahmen der KEYNOTE-146-Studie (Phase Ib/II) untersucht: 108 Patientinnen mit bereits vorbehandeltem, metastatischem Endometriumkarzinom erhielten 20 mg Lenvatinib täglich plus 3‑wöchentlich 200 mg Pembrolizumab in fixer Dosierung bis Krankheitsprogression oder Unverträglichkeit der Medikamente [33]. Die Ansprechrate unter der Kombinationstherapie lag bei 38,3 % (10,6 % komplette Remission und 27,7 % partielle Remission), die mediane Dauer des Ansprechens war bei Data-Cut-off der Studienpublikation noch nicht erreicht; 69 % der Patientinnen mit radiologisch dokumentiertem Ansprechen zeigten im bisherigen Beobachtungszeitraum eine Progressionsfreiheit von ≥6 Monaten. Diese Daten führten im September 2019 zur Zulassung durch die FDA von Pembrolizumab in Kombination mit Lenvatinib zur Behandlung von Patienten mit metastasiertem Endometriumkarzinom, die zumindest eine Vortherapie erhalten hatten und deren Tumoren keine Mikrosatelliteninstabilität oder defiziente Mismatch-repair-Maschinerie aufweisen.

Patientinnen mit rezidiviertem Endometriumkarzinom und nachgewiesener MSI-H/dMMR können im Rahmen der agnostischen Indikation (s. unten) mit Pembrolizumab behandelt werden, sofern bereits eine systemische Therapie erfolgt ist. Bei Endometriumkarzinomen handelt es sich um die Tumorentität mit dem höchsten Anteil (30 %) der Tumoren mit nachweisbar hoher Mikrosatelliteninstabilität (MSI-H) als Folge einer defizienten Mismatch-repair-Maschinerie (dMMR) [34].

In Abb. 2e, f ist das Tumoransprechen einer 59-jährigen Patientin mit lokal rezidiviertem Endometriumkarzinom mittels MRI dokumentiert, die im „Off-label-Use“ eine Kombinationsbehandlung mit den Checkpointinhibitoren Ipilimumab (Anti-CTLA-4-Antikörper) und Nivolumab (Anti-PD1-Antikörper) für 4 Zyklen erhielt. Vorgängig ist die Patientin auf eine Erstlinientherapie mit Cisplatin/Doxorubicin progredient gewesen. Molekularpathologisch konnte im Tumorgewebe eine Hypermethylierung des *MutL Homolog 1*(*MLH1*)-Gen-Promotors als Hinweis auf eine defiziente Mismatch-repair-Maschinerie nachgewiesen werden; 14 Monate nach Abschluss der Behandlung besteht bei der Patientin weiterhin eine komplette Remission der Erkrankung.

### ICI in der Behandlung hämatologischer Neoplasien: klassisches Hodgkin-Lymphom (HL) und primär mediastinales, großzelliges B-Zell-Lymphom (PMBCL)

Eine der höchsten Ansprechraten erzielen ICI interessanterweise beim Hodgkin-Lymphom. Ursächlich liegt diesem klinischen Phänomen eine genetische Veränderung der Hodgkin-Zellen (Amplifikation eines DNA-Segmentes auf dem kurzen Arm von Chromosom 9, 9p24.1) zugrunde, welche zu einer vermehrten Expression von PD-L1 und PD-L2 auf den Lymphomzellen führt [35].

Aufgrund des hohen Heilungspotenzials einer konventionellen Chemotherapie bei Hodgkin-Patienten in der Erstlinienbehandlung wird Nivolumab bei dieser Erkrankung nur selten eingesetzt, vornehmlich in der Behandlung eines zweiten Rezidivs (nach konventioneller Chemotherapie und nach Salvage-Hochdosischemotherapie mit autologer Stammzelltransplantation). Für diese intensiv vorbehandelten Patienten mit zuvor äußerst schlechter Prognose können unter einer Behandlung mit Nivolumab Ansprechrate von 69 % und ein 2‑Jahres-Überleben von 85 % der behandelten Patienten erreicht werden [36].

Bei dem primär mediastinalen, großzelligen B‑Zell-Lymphom (PMBCL) handelt es sich um einen seltenen Subtyp des diffus-großzelligen B‑Zell-Lymphoms. Liegt bei Einsatz einer konventionell dosierten Polychemotherapie (z. B. einer Kombination aus Rituximab/Cyclophosphamid/Adriblastin/Oncovin/Prednison) als Erstlinienbehandlung die Heilungsrate noch bei über 90 %, sinkt diese im Falle einer Therapierefraktärität oder eines Rezidivs beträchtlich mit einem Gesamtüberleben der betroffenen Patienten von lediglich 15 % nach 2 Jahren unter Salvage-Chemotherapie(n) [37]. Ähnlich dem klassischen Hodgkin-Lymphom exprimieren PMBCL vermehrt PD-L1/PD-L2 auf der Zelloberfläche. Ganz ähnlich den Hodgkin-Lymphomen sind genetische Alterationen (z. B. Amplifikationen) auf dem kurzen Arm von Chromosom 9 für die Überexpression von PD-L1/2 verantwortlich. Für Patienten die bereits 2 oder mehr Therapien in der Rezidivsituation erhalten hatten, konnten mit dem Einsatz von Pembrolizumab Ansprechraten von über 45 % erreicht werden, 78 % der Patienten mit dokumentiertem Ansprechen erzielten Remissionen von mehr als 12 Monaten, das mediane Gesamtüberleben lag bei über 31 Monaten in der Studienpopulation [38].

## Agnostische Indikation für Pembrolizumab: Erstmals erfährt ein Krebsmedikament die Zulassung unabhängig vom Ursprungstumor

Für den Anti-PD1-Antikörper Pembrolizumab erließ die FDA im Mai 2017 die Zulassung für Patienten mit soliden Tumoren mit hoher Mikrosatelliteninstabilität (MSI-H) oder defizienter Mismatch-repair-Maschinerie (dMMR), die bereits vorbehandelt waren und für die keine weiteren etablierten Therapieoptionen bestanden. Dies bedeutete zudem die erstmalige Zulassung eines Krebsmedikamentes, basierend rein auf einem Biomarker und nicht wie bisher organbezogen. Dem Einsatz von Pembrolizumab bei Tumoren mit MSI-H/dMMR liegt die Erkenntnis zugrunde, dass gerade diese Tumoren mehrheitlich eine hohe Mutationsrate aufweisen und damit die Wahrscheinlichkeit zur Bildung tumorspezifischer Antigene, sog. Neoantigene, welche in der Folge vom körpereigenen Immunsystem als „fremd“ erkannt werden könnten, steigt.

Die Zulassung basierte auf den „gepoolten“ Ergebnissen mehrerer einarmiger Studien (u. a. Keynote-164 und Keynote-158) an entsprechend vorcharakterisierten Patienten mit insgesamt 15 verschiedenen Tumorentitäten, in denen ein Gesamtansprechen von 39,6 % (davon 7 % mit kompletter Remission) dokumentiert werden konnte. Für 78 % der Patienten mit Tumoransprechen konnte ein progressionsfreies Überleben von mehr als 6 Monaten erreicht werden.

Die Prävalenz des genetischen Markers „MSI-H“ variiert dabei beträchtlich zwischen den einzelnen Tumorentitäten: Für insgesamt 12 Tumorentitäten ließ sich zumindest in 1 % der Tumoren MSI‑H nachweisen, dabei wurde dieses genetische Charakteristikum vergleichsweise häufig für Endometriumkarzinome (31 %), Kolonkarzinome (19 %), Magenkarzinome (17 %) und Rektumkarzinome (5,7 %) nachgewiesen (alle 4 Tumorentitäten sind auch mit dem Lynch-Syndrom assoziiert), hingegen fehlte diese Eigenschaft gänzlich bei uvealen Melanomen, Schilddrüsenkarzinomen und Keimzelltumoren [34]. Doch trotz des Vorliegens von MSI-H/dMMR als möglicher prädiktiver Marker zeigen immer noch 60 % dieser Tumoren kein Ansprechen auf die Behandlung mit Pembrolizumab. Die primären Resistenzmechanismen einer ICI-Behandlung werden aber erst in Anfängen verstanden.

### Immunvermittelte Nebenwirkungen unter Immuncheckpoint-Inhibitortherapie

Die erfolgreiche Einführung der ICI in den onkologischen Alltag bietet Krebspatienten ganz neue Behandlungsmöglichkeiten und Perspektiven in unterschiedlichen klinischen Settings mit zum Teil Langzeitremissionen auch bei bereits weit fortgeschrittenen Tumorerkrankungen. Durch die unspezifische Aktivierung des Immunsystems können die ICI aber auch zu inflammatorischen Reaktionen im Körper führen, sog. immunvermittelten Nebenwirkungen, die prinzipiell jedes Organ im Körper betreffen können.

Die zugrunde liegende Pathophysiologie ist in vielen Fällen noch unklar, wird aber im Zusammenhang mit der Rolle der Immuncheckpoints (CTLA‑4 und PD1/PD-L1) in der Aufrechterhaltung der körpereigenen, immunologischen Homöostase gesehen.

Am häufigsten von immunvermittelten Nebenwirkungen betroffen sind der Gastrointestinaltrakt (z. B. Kolitis), die Leber (Hepatitis), endokrine Drüsen (z. B. Hypophysitis) oder die Haut. Weniger häufig sind neurologische, kardiovaskuläre, pulmonale oder auch muskuloskeletale Nebenwirkungen zu beobachten.

Generell zeigt die bisherige Erfahrung im Einsatz der ICI, dass im Vergleich zu einer Monotherapie die Kombinationstherapie (Anti-CTLA-4 + Anti-PD1) mit einer deutlich höheren Rate an immunvermittelten Nebenwirkungen einhergeht: In einer dreiarmigen, randomisierten Phase-III-Studie für Patienten mit metastasiertem, malignen Melanom traten für die Kombinationsbehandlung (Anti-CTLA-4 + Anti-PD1) höhergradige (≥Grad 3 gemäß der „Common Terminology Criteria for Adverse Events“), immunvermittelte Nebenwirkungen in 59 % auf, während in den Behandlungsarmen mit Monotherapien höhergradige Nebenwirkungen in nur 22 % (Anti-PD1-Antikörper Nivolumab) und 28 % (Anti-CTLA-4-Antikörper Ipilimumab) respektive auftraten [39].

Bei geschätzt 0,3–1,3 % der mit ICI behandelten Patienten kann es auch zu immunvermittelten Nebenwirkungen mit tödlichem Verlauf kommen: Für Anti-CTLA-4-Antikörper sind häufigste dokumentierte Todesfälle mit einer Kolitis assoziiert gewesen, für die Anti-PD1- oder Anti-PD-L1-Antikörper ist das Auftreten einer Pneumonitis, Hepatitis oder auch Neurotoxizität für fatale Verläufe beschrieben [40].

Für das Management der immunvermittelten Nebenwirkungen wurden zwischenzeitlich klare Behandlungsalgorithmen je nach betroffenem Organ entwickelt [41]. Diese basieren in erster Linie auf der Verabreichung von Immunsuppressiva, v. a. Kortikosteroiden. Bei schweren, steroidrefraktären Fällen kommen auch Anti-TNF-α-blockierende Antikörper oder Mycophenolat-Mofetil zum Einsatz. Prospektiv sind die entsprechenden Behandlungsansätze zum Nebenwirkungsmanagement aber bisher noch nicht validiert worden.

Dieses weite Spektrum an Nebenwirkungen der ICI-Therapien mit zum Teil auch fatalen Verläufen benötigt daher häufig die Betreuung durch ein interdisziplinäres Team [40, 42].

## Ausblick

Während der Einsatz von ICIs bis anhin vornehmlich in der palliativen Behandlung weit fortgeschrittener Tumoren untersucht wurde, gehen neuere klinische Entwicklungen dahin, die Wirksamkeit von ICI als Monotherapie oder auch als Kombinationspartner (mit einer konventionellen Chemotherapie, gezielten „targeted“ Therapie oder einer dualen ICI-Therapie) im adjuvanten wie auch neoadjuvanten Setting für unterschiedliche Tumorentitäten in klinischen Studien zu evaluieren.

Die bisher erzielten Erfolge, insbesondere die möglichen Langzeitremissionen, gelten aber noch längst nicht für die Mehrheit der mit ICI behandelten Krebspatienten. Der Fokus der aktuellen Forschung liegt daher verstärkt auf der Untersuchung von Resistenzmechanismen (primäre und sekundäre) unter einer Immuntherapie und Möglichkeiten, diese zu überwinden. Hierzu werden in erster Linie Kombinationstherapien aus einem ICI (meist Inhibitoren des PD1/PD-L1-Immuncheckpoints, da besser verträglich als die Anti-CTLA-4-Antikörper) mit einem Medikament gegen spezifische Zielmoleküle (z. B. gegen LAG3, PSMA, VEGFR 1–3, CDK4/6) in klinischen Studien untersucht.

Weitere offene Fragen ergeben sich im klinischen Alltag durch das Fehlen verlässlicher prädiktiver Marker für eine ICI-Therapie sowie Unklarheiten zur Dauer der Behandlung mit einem ICI, insbesondere im Falle einer dokumentierten kompletten Remission, da in vielen Zulassungsstudien die Behandlungszeit im Falle eines Ansprechens und guter Verträglichkeit nicht begrenzt wurde.

In der Weiterentwicklung und Etablierung der Immuntherapeutika als Kombinationspartner im klinischen Alltag wird aber neben der Verbesserung der Wirksamkeit v. a. auch ein besseres Verständnis der spezifischen, immunvermittelten Nebenwirkungen und deren Management entscheidend sein.
